# Study of 'Redhaven' peach and its white-fleshed mutant suggests a key role of CCD4 carotenoid dioxygenase in carotenoid and norisoprenoid volatile metabolism

**DOI:** 10.1186/1471-2229-11-24

**Published:** 2011-01-26

**Authors:** Federica Brandi, Einat Bar, Fabienne Mourgues, Györgyi Horváth, Erika Turcsi, Giovanni Giuliano, Alessandro Liverani, Stefano Tartarini, Efraim Lewinsohn, Carlo Rosati

**Affiliations:** 1 Consiglio per la Ricerca in Agricoltura, Unità di Ricerca per la Frutticoltura-Forlì (CRA-FRF), via la Canapona 1 bis, 47100 Forlì, Italy; 2 Dept. of Vegetable Crops, ARO Newe Ya'ar Research Center, P.O. Box 1021, 30095 Ramat Yishay, Israel; 3 National Agency for New technologies, Energy and Sustainable Economic Development (ENEA), Trisaia Research Center, S.S. 106 km 419+500, 75026 Rotondella, Italy; 4 University of Pécs, Medical School Department of Pharmacognosy, H-7624 Pécs, Rókus u. 2, Hungary; 5 University of Pécs, Medical School, Department of Biochemistry and Medical Chemistry, H-7624 Pécs, Szigeti út 12, Hungary; 6 ENEA, Casaccia Research Center, Via Anguillarese 301, 00123 Roma, Italy; 7 Dipartimento Colture Arboree, Università di Bologna, via Fanin 42, 40127 Bologna, Italy

## Abstract

**Background:**

Carotenoids are plant metabolites which are not only essential in photosynthesis but also important quality factors in determining the pigmentation and aroma of flowers and fruits. To investigate the regulation of carotenoid metabolism, as related to norisoprenoids and other volatile compounds in peach (*Prunus persica *L. Batsch.), and the role of carotenoid dioxygenases in determining differences in flesh color phenotype and volatile composition, the expression patterns of relevant carotenoid genes and metabolites were studied during fruit development along with volatile compound content. Two contrasted cultivars, the yellow-fleshed 'Redhaven' (RH) and its white-fleshed mutant 'Redhaven Bianca' (RHB) were examined.

**Results:**

The two genotypes displayed marked differences in the accumulation of carotenoid pigments in mesocarp tissues. Lower carotenoid levels and higher levels of norisoprenoid volatiles were observed in RHB, which might be explained by differential activity of carotenoid cleavage dioxygenase (CCD) enzymes. In fact, the *ccd4 *transcript levels were dramatically higher at late ripening stages in RHB with respect to RH. The two genotypes also showed differences in the expression patterns of several carotenoid and isoprenoid transcripts, compatible with a feed-back regulation of these transcripts. Abamine SG - an inhibitor of CCD enzymes - decreased the levels of both isoprenoid and non-isoprenoid volatiles in RHB fruits, indicating a complex regulation of volatile production.

**Conclusions:**

Differential expression of *ccd4 *is likely to be the major determinant in the accumulation of carotenoids and carotenoid-derived volatiles in peach fruit flesh. More in general, dioxygenases appear to be key factors controlling volatile composition in peach fruit, since abamine SG-treated 'Redhaven Bianca' fruits had strongly reduced levels of norisoprenoids and other volatile classes. Comparative functional studies of peach carotenoid cleavage enzymes are required to fully elucidate their role in peach fruit pigmentation and aroma.

## Background

Among Rosaceae, peach (*Prunus persica *L. Batsch) is an appealing model crop, because of its economical value, small genome, rapid generation time and several Mendelian traits (i.e. flesh/leaf/flower color, smooth/fuzzy skin, clingstone/freestone, normal/dwarf growth habit) still to be functionally characterized [1,2]. Peaches are appreciated for their visual, nutritional and organoleptic features, partially contributed by carotenoids, sugars, acids and volatile organic compounds (VOCs), which vary as a function of genetic, developmental and post-harvest factors [[3-5] and references therein]. In particular, carotenoid accumulation in the mesocarp determines the difference between yellow- and white-fleshed genotypes, the latter being generally characterized by a peculiar and more intense aroma. Flesh color is a Mendelian trait (white genotype dominant over yellow [6]), associated with the *Y *locus that has been mapped on the linkage group 1 of the *Prunus *map [7] but which has not been yet functionally characterized from the molecular or enzymatic point of view. Natural mutations, originating flesh color chimera with irregular yellow and white distribution, have long been observed in peach [8].

**Figure 1:**
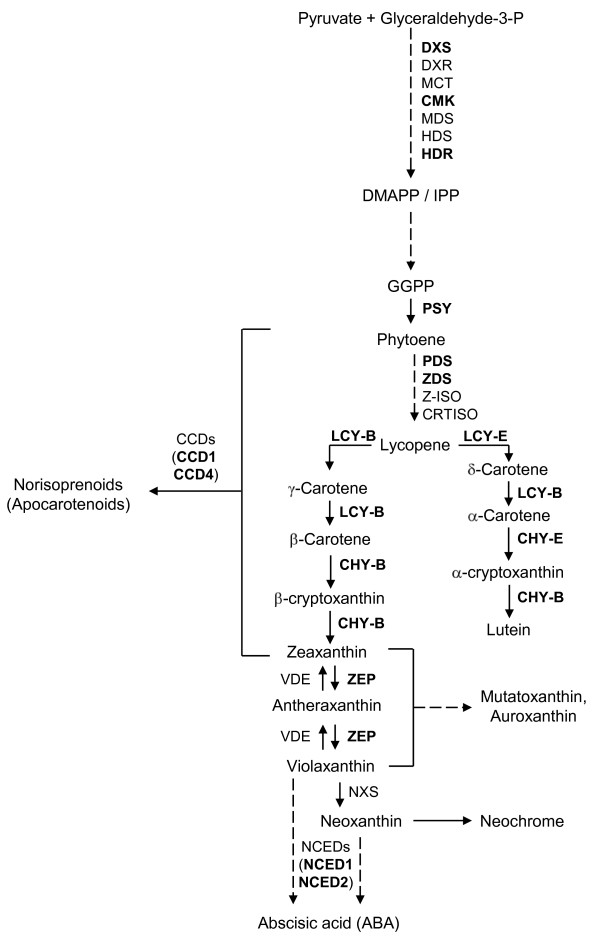
**Schematic representation of isoprenoid and carotenoid pathways in plants**. Enzymes whose encoding gene transcripts were analyzed by RT-qPCR are outlined in boldface. Steps involving multiple enzymes are outlined with dashed arrows. Gene/enzyme acronyms (in alphabetical order): CCD1 and CCD4, carotenoid cleavage dioxygenases 1 and 4; CHY-B, carotene β-hydroxylase; CHY-E, carotene ε-hydroxylase; CMK, 4-(cytidine 5'-diphospho)-2-*C*-methyl-d-erythritol kinase; CRTISO, carotenoid isomerase; DXR, 1-deoxy-d-xylulose 5-phosphate reductoisomerase; DXS, 1-deoxy-d-xylulose 5-phosphate synthase; HDR, 4-hydroxy-3-methylbut-2-enyl diphosphate reductase; HDS, 4-hydroxy-3-methylbut-2-enyl diphosphate synthase; LCY-B, lycopene β-cyclase; LCY-E, lycopene-e-cyclase; MCT, 2-*C*-methyl-d-erythritol 4-phosphate cytidylyltransferase; MDS, 2-*C*-methyl-d-erythritol 2,4-cyclodiphosphate synthase; NCED1 and NCED2, 9-*cis*-epoxycarotenoid dioxygenases 1 and 2; PDS, phytoene desaturase; PSY, phytoene synthase; VDE, violaxanthin de-epoxidase; ZDS, ζ-carotene desaturase; ZEP, zeaxanthin epoxidase; Z-ISO, ζ-carotene isomerase. For a review on the processes and relationships involved in plant VOC biosynthesis pathways, the reader is referred to [57].

Carotenoids are a widespread class of compounds having important functions across living organisms, whose accumulation shows striking phylum- and genotype-specific regulation [9]. Following the formation of the first carotenoid phytoene from the general isoprenoid pathway, the pathway bifurcates after lycopene with respect to the ring type, giving rise to carotenes and xanthophylls with either β-β or β-ε rings (Figure 1, Additional File 1). In addition to their roles in plants as photosynthetic accessory pigments and colorants, carotenoids are also precursors to norisoprenoids (also called apocarotenoids). Norisoprenoids are commonly found in flowers, fruits, and leaves of many plants [10] and possess aromatic properties together with low odor thresholds (e.g., β-ionone), thus having a strong impact on fruit and flower aroma even at low levels [11]. An increasing number of dioxygenase enzymes that specifically cleave carotenoid compounds to form volatile norisoprenoids, abscisic acid (ABA) and regulators of plant growth and development has been characterized. These enzymes have been referred to as carotenoid cleavage dioxygenases (CCDs) and 9-*cis*-epoxycarotenoid dioxygenases (NCEDs) [12] and represent a plant multienzyme family: Arabidopsis has nine CCD/NCED members, of which four have been classified as CCDs (AtCCD1, AtCCD4, AtCCD7 and AtCCD8) and the remaining as ABA-related NCEDs [13]. Functional analysis of CCD enzymes determined that CCD7 and CCD8 are mostly related to the synthesis of norisoprenoid (apocarotenoid) plant hormones, while CCD1 and CCD4 are preferentially involved in volatile production, by using different carotenoid substrates with variable specificity and cleavage site, which probably contributes to the diversity of norisoprenoids found in nature [[14-17] and references therein]. The synthesis of β-ionone, geranylacetone and 6-methyl-5-hepten-2-one in tomato fruits increases 10-20 fold during fruit ripening and these compounds were produced by the activity of *ccd *products [18]. Silencing of *ccd *genes resulted in a significant decrease of the β-ionone content of tomato ripe fruits and petunia flowers [18,19], and increased pigmentation of potato tubers and *Chrysanthemum *flowers [20,21]. CCDs were also implied to be involved in the formation of important apocarotenoid aroma compounds in melon fruit [16]. Furthermore, comparative genetics studies have indicated that carotenoid pigmentation patterns have profound effects on the norisoprenoid and monoterpene aroma volatile compositions of tomato and watermelon fruits [22]. Many norisoprenoids strongly contribute to peach fruit aroma, and their levels increase during fruit ripening [5]. The partial purification and biochemical characterization of β-carotene degrading enzyme(s) from nectarine skin extracts was associated with the formation of C13 norisoprenoids [23]. The study of two NCED-encoding genes from peach showed their differential expression, suggesting a functional relation to ABA formation during fruit ripening [24]. The recent synthesis of specific carotenoid dioxygenase inhibitors [25,26] enables to assess the role of such enzymes *in vivo*, not only in ABA biosynthesis but also in fruit VOC metabolism.

**Figure 2:**
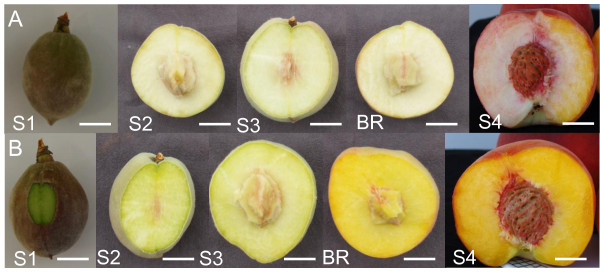
**Peach fruit sampling stages used for this work**. Representative sampled fruits of RHB (A) and RH (B). Bar length (in cm): S1, 3; S2, 3.5; S3, 5; Br, 6; S4, 7. For description of ripening stages, see Methods.

In order to improve our knowledge of carotenoid and VOC biosynthesis in peach fruit and determine the factor(s) controlling carotenoid accumulation in peach flesh, the two cultivars 'Redhaven' (RH; yellow-fleshed) and its white-fleshed bud sport mutant 'Redhaven Bianca' (RHB) [27] were investigated. Carotenoid accumulation, VOC content and transcript levels of relevant carotenoid biosynthetic genes were studied at five stages of fruit development (Figure 2). The effect of the carotenoid dioxygenase inhibitor, abamine SG, on fruit VOC composition at full ripening was also studied.

## Results

### Fruit phenotype during ripening

At S1-S3 stages, whole fruits of RHB (Figure 2A) and RH (Figure 2B) look similar, while the final ripening stages Br and S4, carotenogenesis is well established in RH fruits, and differences in flesh color between yellow-fleshed RH and white-fleshed RHB fruits become dramatic. The yellow pigmentation visible along the suture at full ripening stage (Figure 2A, S4) indicates that RHB is a bud sport chimera mutant, in which the mutation does not involve the L-I apical cell layer, that originates the epidermis and several cells layers at the suture of the ovary wall [28]. The flesh color phenotype has remained stable throughout several cycles of clonal propagation, pointing out the stability of the chimera in RHB (Liverani A., unpublished data). The mutation is transmitted to the progeny in a Mendelian fashion, and is associated to the *Y *locus controlling fruit flesh color. RHB is heterozygous for this locus (*Yy*), originating either 1:3 of yellow- to white-fleshed seedlings when selfed or 1:1 of yellow- to white-fleshed progenies when crossed with yellow (*yy*) peach accessions (Liverani A., unpublished data). The yellow strip is not observed in white-fleshed RHB progenies (Liverani A., unpublished data), because the gametes does not originate from the L1 cell layer but from the fully-mutated L2 layer. The major fruit quality traits and skin color parameters of the two cultivars were also measured, showing statistically significant differences only for soluble solids content (Additional File 2).

### Carotenoid composition of RHB and RH fruits

**Figure 3:**
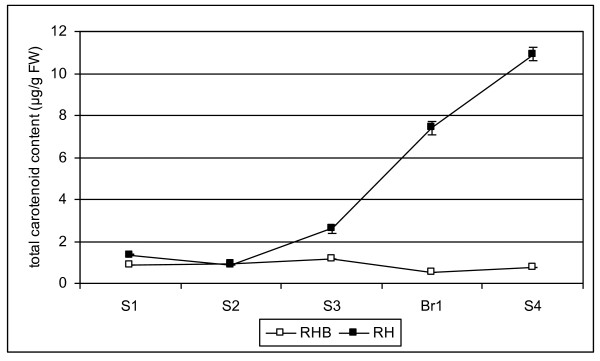
**Carotenoid accumulation in RHB and RH mesocarp during fruit ripening**. RH: solid black squares. RHB: open squares. Total carotenoid levels ± SD are in μg/g fresh weight.

In carotenoid-containing fruits, the massive biosynthesis of such compounds is generally associated with late ripening stages and plastid transition from chloroplasts into chromoplasts. At early ripening stages S1 and S2, fruits of RHB and RH had similar total carotenoid levels and accumulated only a few carotenoid compounds, dominated by the presence of lutein and β-carotene (Figure 3; Table 1). From the S3 stage, RH mesocarp accumulated increasing amounts of carotenoids that peaked at the S4 stage to provide the solid yellow flesh color, while carotenoid content in RHB flesh remained low (Figure 3). Detailed HPLC analysis revealed the presence of specific carotenoid compounds in the two genotypes (Table 1), some of which rather uncommon and present in RH only, whose main chemical structures are illustrated (Additional File 1). Until stage S3 (RH) and Br (RHB), lutein and its (*Z*)-isomers were the major carotenoids in fruits of both genotypes, accounting for over 50% of the total carotenoid pool. Other major carotenoids at early stages were β-carotene, relatively abundant in fruits of both cultivars, and neochrome epimers, which accumulated only in RH fruits. From stage S3, not only did RH fruits have higher carotenoid levels, but also a range of carotenoid compounds much wider than RHB (Table 1). At full ripening S4 stage, xanthophylls made up the majority of carotenoids - zeaxanthin was the main carotenoid in RHB, while antheraxanthin, luteoxanthin and zeaxanthin were the most abundant compounds in RH fruits (Table 1).

**Table 1:** Carotenoid composition of RH and RHB fruits during ripening.

Stage	Compound	Amount	
		
		RH	RHB
S1	Lutein isomers	830	457
	β-carotene	178	310
	Neochrome epimers	141	n.d.
	(3-hydroxy)-5,6-epoxy-5,6-dihydro-β-ionone + (3-hydroxy)-5,6-epoxy-5,6-dihydro-10'-apo-β-carotenal (tent.)	76	n.d.
	β-cryptoxanthin	64	68
	Violaxanthin	56	n.d.
	Zeaxanthin	n.d.	50
S2	Lutein isomers	577	485
	Neochrome epimers	114	n.d.
	β-carotene	109	313
	(3-hydroxy)-5,6-epoxy-5,6-dihydro-β-ionone + (3-hydroxy)-5,6-epoxy-5,6-dihydro-10'-apo-β-carotenal (tent.)	54	n.d.
	Violaxanthin	23	n.d.
	β-cryptoxanthin	21	48
	Zeaxanthin	n.d.	54
	Zeinoxanthin (tent.)	n.d.	27
	β-cryptoflavin	n.d.	25
S3	Lutein isomers	1394	690
	Neochrome epimers	382	n.d.
	β-carotene	292	330
	Violaxanthin isomers	253	n.d.
	Luteoxanthin epimers	137	n.d.
	(3-hydroxy)-5,6-epoxy-5,6-dihydro-β-ionone + (3-hydroxy)-5,6-epoxy-5,6-dihydro-10'-apo-β-carotenal (tent.)	77	n.d.
	Auroxanthin	49	n.d.
	Zeaxanthin	n.d.	77
	β-cryptoflavin	n.d.	34
	Zeinoxanthin (tent.)	n.d.	31
Br	Luteoxanthin epimers	2506	n.d.
	(9*Z*)-lutein-5,6-epoxide isomers	942	n.d.
	β-carotene	912	219
	Violaxanthin isomers	741	n.d.
	Lutein isomers	693	248
	Zeaxanthin isomers	523	27
	Neochrome epimers	437	n.d.
	β-cryptoxanthin	208	23
	Mutatoxanthin epimers	148	n.d.
	Latochrome (tent.)	141	n.d.
	(all-*E*)-neoxanthin	89	n.d.
	Unidentified compound	67	n.d.
	(*Z*)-auroxanthin	7	n.d.
S4	Antheraxanthin isomers	2391	n.d.
	Luteoxanthin isomers	2304	n.d.
	Zeaxanthin isomers	1539	427
	Mutatoxanthin epimers	972	95
	β-cryptoxanthin	742	31
	β-carotene	583	n.d.
	(all-*E*)-violaxanthin	491	n.d.
	Neochrome epimers	459	n.d.
	Phytofluene	389	20
	Lutein isomers	480	150
	Neoxanthin isomers	275	n.d.
	Latochrome epimers (tent.)	183	n.d.
	Unidentified compound	76	n.d.
	(3-hydroxy)-5,6-epoxy-5,6-dihydro-β-ionone + (3-hydroxy)-5,6-epoxy-5,6-dihydro-10'-apo-β-carotenal (tent.)	55	8
	ζ-carotene	n.d.	20

Average values are listed in descending order with respect to RH composition and expressed in ng/g fresh weight. PDA λ: 450 nm. tent.: tentative identification. n.d.: not detectable.

### Gene expression analyses

Relative transcript levels of three genes involved in isoprenoid metabolism [1-deoxy-d-xylulose 5-phosphate synthase (*dxs*), 4-(cytidine 5'-diphospho)-2-*C*-methyl-d-erythritol kinase (*cmk*) and 4-hydroxy-3-methylbut-2-enyl diphosphate reductase (*hdr*)] and twelve genes involved in carotenoid biosynthesis and cleavage [phytoene synthase (*psy*), phytoene desaturase (*pds*), ζ-carotene desaturase (*zds*), lycopene β-cyclase (*lcy-b*), lycopene ε-cyclase (*lcy-e*), carotene β-hydroxylase (*chy-b*), carotene ε-hydroxylase (*chy-e*), zeaxanthin epoxidase (*zep*), two carotenoid cleavage dioxygenases (*ccd1 *and *ccd4*), and two 9-*cis*-epoxycarotenoid dioxygenases (*nced1 *and *nced2*)] (Figure 1) were measured in RH and RHB mesocarp at S1, S2, S3, Br and, S4 stages by reverse transcription quantitative real-time PCR (RT-qPCR).

**Figure 4:**
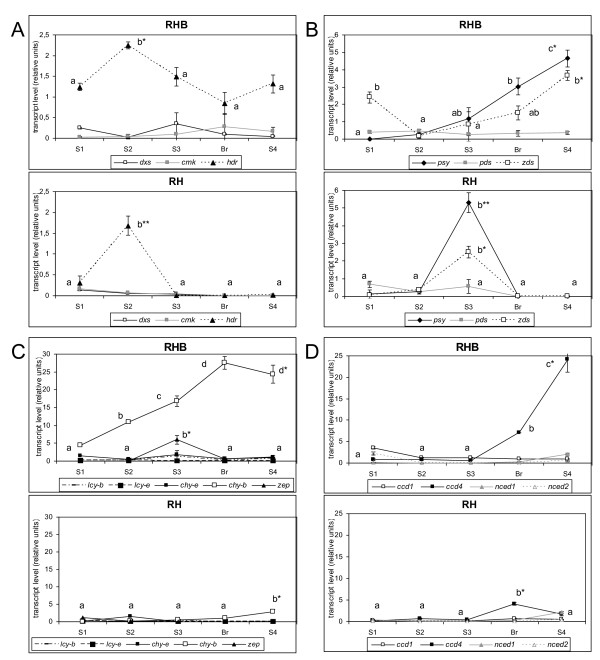
**Expression patterns of carotenoid-related genes during ripening of RHB and RH fruits**. Relative average gene transcript levels ± SD are given, following normalization with *rps28 *values. A: isoprenoid genes [*cmk*, 4-(cytidine 5'-diphospho)-2-*C*-methyl-d-erythritol kinase; *dxs*, 1-deoxy-d-xylulose 5-phosphate synthase; *hdr*, 4-hydroxy-3-methylbut-2-enyl diphosphate reductase]. B: early carotenoid genes (*pds*, phytoene desaturase; *psy*, phytoene synthase; *zds*, ζ-carotene desaturase). C: other carotenoid genes (*chy-b*, carotene β-hydroxylase; *chy-e*, carotene ε-hydroxylase; *lcy-b*, lycopene β-cyclase; *lcy-e*, lycopene-e-cyclase; *zep*, zeaxanthin epoxidase). D: dioxygenase-related genes (*ccd1 *and *ccd4*, carotenoid cleavage dioxygenases 1 and 4; *nced1 *and *nced2*, 9-*cis*-epoxycarotenoid dioxygenases 1 and 2). For each gene, different letters indicate significant differences among mean values from different stages (*: *p *≤ 0.05; **: *p *≤ 0.01).

The isoprenoid pathway genes, *dxs *and *cmk*, had very low transcript levels throughout fruit development in both cultivars. *hdr *showed a sharp peak of expression at the S2 stage which declined at later stages in the yellow-fleshed RH, while its expression remained high in RHB (Figure 4A). Similarly, early carotenoid pathway genes *psy *and *zds *showed a peak at the S3 stage in RH, while in RHB these transcripts showed a constant increase until the S4 stage (Figure 4B). Among later pathway genes (*lcy-b *, *lcy-e*, *chy-b*, *chy-e *and *zep*), only *chy-b *was strongly up-regulated in RHB (Figure 4C), while, *ccd *and *nced *expression was generally low in both genotypes, with the exception of *ccd4*, which was significantly up-regulated in RHB at late ripening stages, its transcript level being 13-fold higher than that in RH at the S4 stage (Figure 4D).

Hierarchical clustering analysis (HCA) of gene expression data clustered the ripening stages consistently with their chronological order in joint analysis of both genotypes (Additional File 3A) and in RH alone (Additional File 3B). In RH, a clear co-regulation of genes encoding enzymes closely positioned in the pathway (*dxs *and *cmk*; *psy*, *pds *and *zds*; *lcy-b *and *lcy-e*; *nced1*, *ccd1*, *ccd4 *and, surprisingly, *chy-b*) was observed (Additional File 3C). Instead, in the RHB mutant the majority of these co-regulation clusters was broken, with the exception of *ccd4 *and *chy-b *genes which remained co-regulated (Additional File 3C).

### VOC analyses

Levels of different VOCs were studied in RHB and RH during fruit ripening by GC-MS. In total, 41 VOCs were detected, assigned to aromatic and branched chain amino acid-related, fatty acid-related, furan-related, lactone, monoterpene and norisoprenoid classes, quantified and underwent further analyses (Table 2).

**Table 2:** VOC composition of fruits of RH and RHB at different ripening stages.

						RH					RHB		
				
Class	Compound	Id.	RI	S1	S2	S3	Br	S4	S1	S2	S3	Br	S4
Aromatic aa-related	Benzaldehyde	MS, KI, Std	960	16.7	4.4	461.2	199.1	72.6	11.2	0.2	223.3	242.7	49.7
	Benzenacetaldehyde	MS, KI, Std	1042	0.4	0.3	18.9	30.2	9.9	0.3	n.d.	18.1	13.1	3.8
	Benzoic acid	MS, KI	1164	1.1	0.3	69.5	46.4	39.7	1.0	0.1	35.3	49.6	34.8
	4-vinylphenol	MS, KI	1219	n.d.	0.1	21.1	16.7	14.8	n.d.	n.d.	11.8	29.3	19.8
	Chavicol	MS, KI	1253	n.d.	n.d.	0.7	6.7	40.0	n.d.	n.d.	n.d.	7.6	10.5
	Eugenol	MS, KI, Std	1358	n.d.	n.d.	12.6	85.6	158.4	n.d.	n.d.	6.4	71.0	68.4
	Vanillin	MS, KI, Std	1394	0.2	n.d.	10.5	13.6	21.5	1.4	n.d.	11.1	16.5	13.7
Branched chain aa-related	*Iso *valeric acid	MS, KI	828	n.d.	n.d.	115.2	93.3	71.9	n.d.	n.d.	110.1	77.4	28.4
Fatty acid-related	2-hexen-1-ol-*E*	MS, KI	854	0.8	0.1	123.7	29.7	8.2	0.5	10.0	82.3	31.8	12.7
	3-hexen-1-ol-acetate-*Z*	MS, KI	1005	n.d.	n.d.	7.7	11.0	9.7	n.d.	3.3	4.6	10.0	7.8
	2-hexen-1-ol acetate-*E*	MS, KI	1015	n.d.	n.d.	5.2	31.7	9.7	n.d.	0.3	n.d.	22.8	n.d.
	Dodecane	MS, KI	1189	n.d.	n.d.	25.2	41.7	28.6	n.d.	0.3	27.6	53.1	26.0
	Dodecanoic acid	MS, KI	1556	n.d.	n.d.	41.6	195.0	162.8	n.d.	n.d.	8.7	24.5	19.0
	Tetradecanoic acid	MS, KI	1758	n.d.	n.d.	22.6	42.0	46.2	n.d.	n.d.	9.1	29.8	10.0
	Unknown Fatty acid-rel.-1		1957	0.4	0.4	286.7	441.3	451.0	0.5	0.1	214.6	397.2	238.6
	Unknown Fatty acid-rel.-2		2103	0.3	0.1	69.6	39.5	3.1	0.2	0.2	100.8	102.0	14.3
	Methyl linoleate	MS, KI	2127	0.3	0.4	125.3	160.8	206.8	0.6	0.1	123.8	209.5	161.6
Furan-related	2.5-Furandione	MS, KI	833	3.2	12.2	2007.3	2195.3	3009.0	3.3	n.d.	1668.4	3930.2	2313.2
	3-methyl-2.5-furandione (put.)	MS	941	0.1	0.2	389.9	387.9	197.1	0.1	0.1	438.9	940.4	230.2
	dihydro-2.5-furandione (put.)	MS	1022	0.4	0.4	144.9	210.7	278.8	0.3	n.d.	131.9	208.4	278.9
	3.4-dimethyl-2.5-furandione (put.)	MS	1038	n.d.	n.d.	13.4	11.4	4.6	n.d.	n.d.	17.2	47.3	41.6
	Unknown Furan-related		1110	n.d.	n.d.	19.6	25.8	18.8	n.d.	n.d.	8.8	20.4	12.6
Lactones	γ-hexalactone	MS, KI	1043	n.d.	n.d.	16.5	31.2	119.5	n.d.	0.1	4.0	12.7	81.5
	δ-deca-2.4-dienolactone (put.)	MS	1453	n.d.	n.d.	2.6	2.1	27.8	n.d.	6.1	n.d.	n.d.	5.2
	δ -decalactone	MS, KI	1493	n.d.	n.d.	10.7	1.8	91.0	n.d.	n.d.	n.d.	n.d.	17.3
Monoterpenes	Linalool	MS, KI. Std	1090	n.d.	n.d.	7.2	14.9	101.8	n.d.	n.d.	n.d.	n.d.	21.8
	Carvone	MS, KI	1244	n.d.	n.d.	n.d.	3.0	n.d.	n.d.	0.1	1.1	n.d.	n.d.
	8-Hydroxylinalool	MS, KI	1336	n.d.	n.d.	n.d.	n.d.	n.d.	n.d.	n.d.	n.d.	n.d.	n.d.
Norisoprenoids	3-hydroxy-β-damascone (put.)	MS	1618	n.d.	n.d.	24.8	65.7	116.8	n.d.	n.d.	69.9	221.8	258.0
	Unknown norisoprenoid-1		1658	n.d.	n.d.	1.0	11.5	31.3	n.d.	1.3	15.0	48.0	83.1
	3-hydroxy-5.6-epoxy-β-ionone	MS, KI	1683	0.1	n.d.	14.1	12.1	18.6	n.d.	n.d.	27.1	52.3	66.1
	4-hydroxy-3.5.6-trimethyl-4-(3-oxo-1-butenyl)-2-cyclohexen-1-one. (put.)	MS	1785	0.1	n.d.	43.4	60.4	144.8	0.1	n.d.	213.4	259.5	410.2
	Unknown norisoprenoid-2		2220	n.d.	0.1	183.9	211.7	221.4	n.d.	0.3	112.3	183.0	87.1
	Unknown norisoprenoid-3		2244	n.d.	0.1	248.1	282.1	271.3	n.d.	0.1	157.8	258.9	125.2
Others	1H-pyrazole (put.)	MS	1036	n.d.	n.d.	110.7	120.5	74.3	n.d.	0.3	133.4	284.9	60.6
	Pentanoic acid-4-oxo (put.)	MS	1143	n.d.	0.1	28.7	18.8	12.2	n.d.	n.d.	23.8	38.0	9.8
	Salicylic acid (put.)	MS	1294	0.3	n.d.	74.6	98.0	74.5	0.2	n.d.	82.5	120.8	50.6
	2-propanoic acid 3-phenyl-*E *(put.)	MS	1622	2.0	2.3	457.1	362.7	263.5	1.3	n.d.	366.7	463.0	124.5
	Unknown-1		1829	0.1	0.2	118.0	57.0	7.5	0.2	0.1	120.6	122.0	6.5
	Unknown-2		1906	n.d.	0.1	120.4	232.5	323.4	n.d.	0.1	99.5	243.0	179.5
	Unknown-3		1894	0.6	0.5	212.1	287.1	236.2	0.5	0.1	178.9	337.1	94.5

Average data of 4 to 8 replicates. Values are in ng/g fresh weight. Id.: identification method (MS, Mass Spectrometry; KI, Kovacs Index; Std, standard compound data). Positive compound identification was obtained by matching both MS and KI or Standard compound data. Otherwise, putative (put.) best compound is listed. RI: retention index. aa: amino acid. n.d.: not detectable.

**Figure 5:**
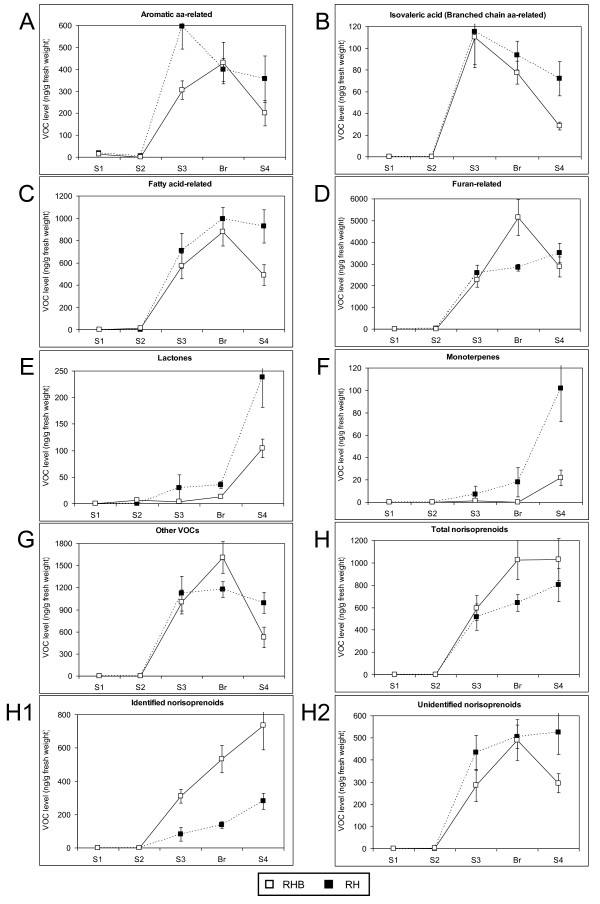
**VOC content in RHB and RH mesocarp during fruit ripening**. RH: solid black squares. RHB: open squares. Developmental patterns of the various VOC classes were obtained by summing the levels of compounds from Table 2. Levels ± SD are in ng/g fresh weight. A: aromatic amino acid-related. B: branched chain amino acid-related. C: fatty acid-related. D: furan-related. E: lactones. F: monoterpenes. G: other VOCs. H: total norisoprenoids. H1: identified norisoprenoids.H2: unidentified norisoprenoids.

The two genotypes had a similar, ripening-associated accumulation of total VOCs starting from the S3 stage, while early S1 and S2 stages were characterized by very low volatile content (Additional File 4). Detailed analysis pointed out differences in the accumulation of the distinct VOC pools in the two genotypes (Figure 5). Furan-related compounds accumulated at the highest levels in both genotypes, followed by norisoprenoids and fatty acid-related compounds, whose maximum levels were about 5-fold lower than those of the furans (compare Figures 5D, C and 5H). The other classes accumulated at lower absolute levels, with maxima in the range of hundreds of μg/g fresh weight. The two genotypes displayed similar ripening-associated patterns for aromatic and branched chain amino acid-, fatty acid-, and furan-related classes, with a peak at the S3/Br stages and a more or less pronounced decline at later ripening stage(s) (Figures 5A-D). Total lactone and monoterpene contents displayed a different pattern, with a strong up-regulation only at final S4 ripening stage in the two cultivars (Figure 5E-F). At the S4 stage, all the six above-mentioned VOC classes had higher levels in RH fruits (Figure 5A-F).

A remarkable exception was the norisoprenoid pool, which accumulated in RHB fruits at levels higher than those of RH from S3 stage on. Norisoprenoid pattern in RHB fruits peaked at Br and was constant through S4 stage, while in RH fruits it displayed a linear increase from S3 stage (Figure 5H). In particular, the three identified norisoprenoids 3-hydroxy-5,6-epoxide-β-ionone, 3-hydroxy-β-damascone and 4-hydroxy-3,5,6-trimethyl-4-(3-oxo-1-butenyl)-2-cyclo-hexen-1-one were responsible for the higher total norisoprenoid levels in fruits of RHB, with an almost 3-fold difference at the S4 stage (Figure 5 H1), when the typical floral scent of white-fleshed peach fruits reaches its maximum. At any ripening stage, the level of each identified norisoprenoid compound was always higher in the white-fleshed RHB than in RH (Additional File 5). Instead, the less prominent unidentified norisoprenoids had a similar accumulation pattern in the two genotypes, with higher levels in RH fruits (Figure 5 H2).

**Figure 6:**
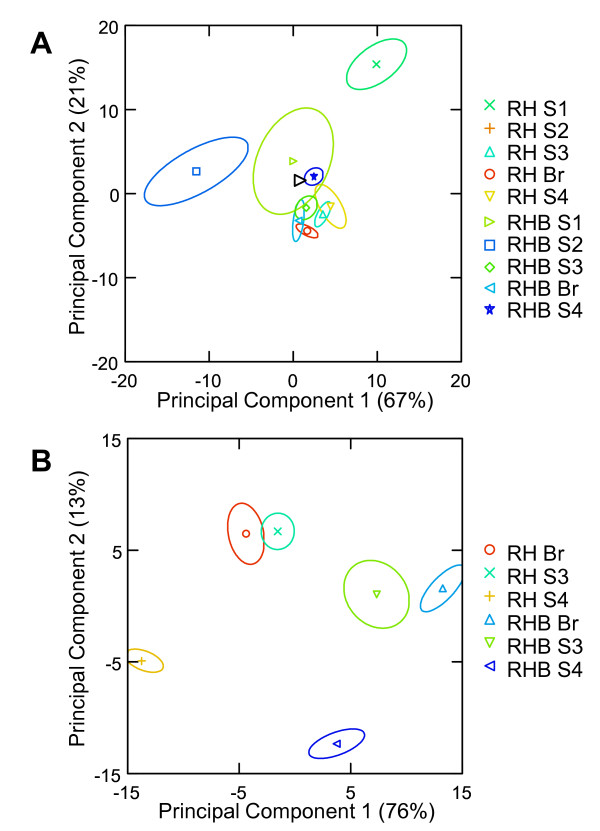
**Principal component analysis of GC-MS data from RHB and RH genotypes**. The various stages are represented with different symbols. The variance explained by each principal component is represented within parentheses. A: PCA of all samples (5 stages). B: PCA of late ripening stages S3, Br and S4.

PCA was performed on the whole GC-MS dataset (41 major VOCs) to provide a more intuitive visualization of data and to discriminate the different ripening stages in the two varieties with respect to VOC composition. A preliminary PCA analysis was carried out including all five stages, and resulted in a poor separation of most samples (Figure 6A), with the exception of RHB-S1, RH-S2, RH-S3 and RH-Br. Principal components 1 and 2 explained 67% and 21% of the total variability, respectively (Figure 6A). A narrower analysis was then carried out by excluding the S1 and S2 samples, which allowed the complete discrimination of the six late ripening samples of both genotypes (Figure 6B). In this closer analysis, the new calculated principal components 1 and 2 accounted for 76% and 13% of the total variability, respectively (Figure 6B).

### Effect of a carotenoid dioxygenase inhibitor on VOC production

**Figure 7:**
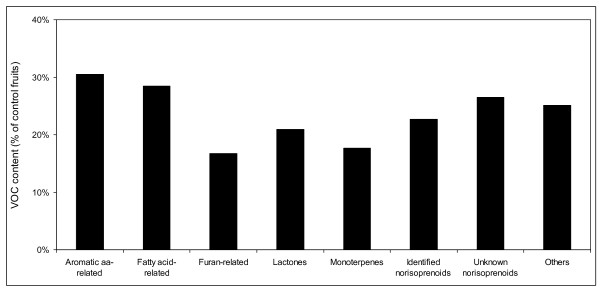
**Effect of abamine SG treatment on VOC content in RHB fruits**. Levels of distinct VOC classes in abamine SG-treated fruits are shown as percentage of those in control fruits.

The effect of abamine SG treatment was assessed in RHB ripe fruits, injected once a week from the S3 stage. As expected, abamine SG injection resulted in a drastic reduction of the levels of both identified and unknown norisoprenoids (Figure 7). Unexpectedly, this treatment also down-regulated the content of the other VOCs. The strongest reduction was observed for furan-related, monoterpene and lactone pools, while the total content of aromatic amino acid- and fatty acid-related compounds was the least affected by abamine SG treatment (Figure 7).

## Discussion

Peach fruits contain carotenoid compounds with significant antioxidant capacity and claimed beneficial health effects. The enzymatic cleavage of these compounds results in the production of volatile norisoprenoids (apocarotenoids). Our study investigated the expression of carotenoid genes, the carotenoid content and the volatile composition in the wild type yellow-fleshed RH and its white-fleshed RHB during fruit ripening.

Key regulatory steps and regulation mechanisms controlling isoprenoid and carotenoid flux in many species, also through biotechnology-based approaches to carotenoid manipulation, have been extensively reviewed [9,29]. Differential accumulation of carotenoids in RH and RHB became evident at stage S3, and reached its maximum at stage S4, when RH fruits accumulated approximately 10-fold more carotenoids than RHB (Figure 3), composed mainly of β-ring carotenoids (Table 1). Accordingly, the two cultivars showed strikingly different developmental patterns of expression of several isoprenoid and carotenoid genes (Figure 4). However, several differential regulation events appeared to be the *effect*, rather than the *cause*, of the differential carotenoid accumulation. For instance, in RHB the up-regulation of *hdr*, *psy*, and *zds *genes at late (Br and S4) stages appears to be the result of a feed-back repression by either the lower carotenoid levels in this genotype or their cleavage products, acting on the transcript levels of these genes. Examples of feed-back regulation of carotenoid gene transcripts have been described in plants where carotenoid biosynthesis has been altered through the use of inhibitors or metabolic engineering [30-32]. A similar higher expression of early carotenoid genes was found in a white-fleshed apricot cultivar with respect to an orange-fleshed genotype [33], possibly suggesting common regulation mechanisms of early carotenoid gene expression based on feedback regulation mediated by carotenoid end products. The generally low expression at late Br/S4 stages of the studied carotenoid genes in the yellow-fleshed RH might point out a control of metabolite flux based on steady-state gene/enzyme expression rather than up- or down regulation of transcription.

On the other hand, in RHB fruits the strong up-regulation at late stages of *chy-b *and *ccd4 *genes is negatively correlated with the accumulation of β-ring carotenoids, and positively correlated with that of identified β-ring norisoprenoids. The biochemical function of the CHY-b and CCD4 gene products is compatible with the observed phenotype of RHB: CHY-b funnels carotenoids into the β-xanthophyll branch, producing substrates cleaved by carotenoid dioxygenases, including CCD4. Furthermore, *chy-b *is a negative regulatory step in several plant systems: inhibition of its expression through transgenosis or natural genetic variation results in higher β-carotenoid levels in potato tubers and maize endosperm [34,35]. Similarly, the levels of *ccd4 *transcript negatively correlate with carotenoid levels (see below).

Aubert et al. [4,5] described the presence of several C13 norisoprenoids, mainly derived from the cleavage of β-ring xanthophylls like in the present study, in yellow- white-fleshed nectarines. Unlike RH and RHB, the two cultivars were not isogenic, but similarly to the present case, the white-fleshed nectarine 'Vermeil' had higher norisoprenoid contents than the yellow-fleshed 'Springbright'. However, in two other studies, the β-ionone levels detected in a number of white-fleshed genotypes were lower than those of several unrelated yellow-fleshed accessions [36,37]. Such examples show that flesh color *per se *is not sufficient to determine the levels of norisoprenoid VOCs, confirming that volatile composition strongly depends on genetic factors other than carotenoid levels. In our study, RHB ripe fruits had higher norisoprenoid content than that of RH, derived from carotenoid cleavage. As a likely consequence of the redirection of metabolite flux towards the synthesis of nor/isoprenoid compounds in RHB fruits, the levels of all other VOC pools were lower than those in RH fruits.

Among aroma-related carotenoid dioxygenases, both CCD1 and CCD4 enzymes cleave carotenoids at the 9,10 and 9',10' positions and have a major role in the formation of β-ionone and other fruit and flower norisoprenoids [18,19,38-41]: the β-carotene degradation displayed by yellow nectarine skin extracts [23] most likely corresponds to CCD1 and/or CCD4 activity. Compared with CCD1s, the CCD4 enzymes were characterized more recently from several crops [40-43], and shown to have a major impact on organ pigmentation: *ccd4 *expression was higher in white-fleshed potato and in white-flowered *Chrysanthemum *genotypes than in their respective yellow-pigmented counterparts, and RNAi-mediated knockout boosted carotenoid accumulation [20,21]. The small CCD4 family contains at least two forms of genes with different structure, expression patterns and genome position, and their encoded enzymes show different substrate specificity [41,42]. The existence of plastid target peptides and the demonstrated plastid localization of CCD4 enzymes [41] allow these enzymes direct access to the carotenoid substrates, suggesting that they start the carotenoid degradation and norisoprenoid synthesis pathway. On the other hand, the lack of correlation between the patterns of *ccd1 *expression (Figure 4D) and norisoprenoid content (Figure 5H, H1 and H2) in the flesh of both cultivars reflects a situation common to other fruits like grape, melon, and tomato [16,18,39]. This is a likely consequence of the different localization of CCD1s and their substrates, since carotenoids are accumulated in plastids but known CCD1s lack plastid transit peptides [14,38], and/or substrate preference, since each carotenoid dioxygenase can accept different carotenoids. Our data suggest that because of their different subcellular localization, CCD1s only contribute to volatile production, while CCD4s are likely to control also carotenoid degradation. As to other dioxygenase-encoding genes studied, *nced1 *and *nced2 *expression patterns were up-regulated during ripening (Additional File 3, D), in agreement with the reported physiological increase of ABA content at late fruit ripening stages [24], with a profile similar to that of ethylene-related *accS *and *accO *genes (data not shown).

The transcriptional control of peach norisoprenoid content by specific dioxygenase genes is similar to that of fatty acid pathway-related genes and enzymes, whose expression patterns generally show a strong positive correlation with those of the corresponding volatiles [44,45]. The strong down-regulation of norisoprenoids in ripe RHB fruits treated with abamine SG, a carotenoid dioxygenase inhibitor applied at late steps of fruit development, confirmed the hypothesis that their production is mediated by carotenoid cleavage dioxygenases. Although abamine SG was reported to be a specific carotenoid cleavage dioxygenase and ABA biosynthesis inhibitor, more potent than the previously synthesized abamine [25,26], the authors did not investigate pathways other than that leading to ABA formation under stress conditions. Therefore, the observed unexpected reduction of other VOC classes could be due to the non-specific inhibition of other dioxygenase enzymes active in different aroma pathways, suggesting that abamine SG is a useful tool to investigate VOC metabolism.

If CCD4 is confirmed to be the major factor responsible for carotenoid degradation, then the mutation that generated the RHB cultivar is likely to be a gain-of-function mutation, restoring *ccd4 *function. This is compatible with the dominant nature of the white flesh phenotype over the yellow one. Since the mutation originating RHB is associated with the *Y *locus (cf unpublished data of progeny tests, Results section), a crucial step of future research is to determine whether the *Y *locus, mapped on LG1, is linked to the *ccd4 *gene. The recently released draft of the peach genome sequence (http://www.rosaceae.org/peach/genome) will enable future comprehensive research towards the functional characterization of the peach CCD/NCED family, required to confirm the presented data on *ccd4 *and to elucidate the function of each member in different peach genotypes.

## Conclusions

This study presented a comprehensive molecular and biochemical research of the carotenoid and VOC metabolism in peach fruit, driven by developmental and genetic cues, and pointed out the central role of carotenoid cleavage dioxygenases, namely the product of *ccd4*, in flesh color and peach aroma formation. By taking advantage of a wild type-mutant system contrasted in fruit flesh color, we provided new information for understanding the mechanisms controlling carotenoid biosynthesis, flesh color and volatile content in peach, which could be useful for the optimization of these important fruit quality traits. The completion of peach genome will improve existing databases [[46], http://www.rosaceae.org] and boost genetic and molecular research to confirm the role and interactions of carotenoid cleavage dioxygenases with other factors controlling fruit pigmentation and aroma, stimulating new comprehensive research on peach fruit quality traits [47,48].

## Methods

### Plant material and sampling

Fruits of cv RH and RHB were harvested from trees grown in an experimental field located near Forlì (Po Valley, Italy; 44.161° N, 12.088° E) at five different ripening stages according with the growth curve (Figure 2): S1 (about 35 days after pollination, dap), S2 (about 50 dap), S3 (about 90 dap), Breaker (Br; about 115 dap), and S4 (about 122 dap) (Figure 2). For molecular and biochemical analyses, two replicates of four representative fruits of each stage were sampled, peeled, cut into 0.5-cm slices and the mesocarp was immediately frozen in liquid nitrogen and stored at -80 °C. In RHB, the tissues near the suture were discarded since were not affected by the mutation originating the white-fleshed phenotype. For visual and organoleptic quality scoring (Additional File 2), S4 fruits underwent the following measurements immediately after harvest: flesh firmness (FTA penetrometer, Turroni, Italy, equipped with an 8-mm plunger tip), soluble solid content (Smart1 automatic refractometer; Atago Co., Tokyo, Japan), titratable acidity (titration of 10 ml peach juice with NaOH 0.2 M until pH 8.2), skin color (Chroma Meter CR-200 reflectance colorimeter; Minolta Camera Co., Osaka, Japan).

For abamine SG treatments, 100 μl of an abamine SG solution (0,1 mM in DMSO) were injected with a hypodermical needle in different points of RHB fruits once a week from S3 stage until S4. DMSO-injected fruits were taken as controls. Mesocarp samples were collected at S4 stage as previously described, and used for GC-MS analyses.

### Molecular procedures

Experiments complied with the MIQE guidelines [49], and relevant information is contained in the manuscript. Total RNA (two independent replicates for each sample) was isolated from frozen fruit tissue as reported [50]. First strand cDNA was synthesized from 1 μg total RNA in 30 μL with oligo-d(T)17 and Superscript III (Invitrogen, Milan, Italy), according to manufacturer's instructions. cDNA concentration in the RT mix was quantified using a ND-1000 UV spectrophotometer (Nanodrop, Wilmington, USA). First strand cDNA (10 ng) was used as template for RT-qPCR assays, carried out with primers for *rps28 *and carotenoid genes. The *rps28 *gene was chosen as reference gene, based on preliminary tests (data not shown). All reactions were performed using the Applied Biosystems 7900HT Real Time PCR system and Platinum^® ^SYBR^® ^Green RT-qPCR SuperMix-UDG plus ROX (Applera Italia, Monza, Italy), following the manufacturer's procedures. No template and no amplification controls were included in each experiment.

Three RT-qPCR runs were carried out for each cDNA and gene to serve as technical replicates. PCR conditions were: 5 min at 95°C, followed by 45 cycle at 95°C for 15 s and at 58°C for 60 s. At the end of the PCR, melting curve analysis was carried out to check for the presence of the correct amplicons only. Relative transcript abundance was quantified using the relative standard curve method described in the ABI PRISM 7900 HT manual, and the data was normalized against the quantity of the reference *rps28 *transcript. Serial 10-fold dilution of each gene fragment were used to calculate the standard curve and measure the amplification efficiency for each target and reference gene. Sequences of peach *dxs*, *cmk*, *hdr*, *psy*, *pds*, *zds*, *lcy-b*, *lcy-e*, *chy-b*, *chy-e*, *zep*, *ccd1*, *nced1 *and *nced2 *genes, and the reference gene *rps28*, were obtained from NCBI database and [51]. A 1.84-kb putative peach *ccd4 *gene was retrieved from the recently published peach draft genome [52] (Phytozome v6.0 search tool at http://www.phytozome.org/peach) by searching with a *Malus ccd4 *sequence (EU327777). The identified peach *ccd4 *coding sequence share 80% identity with its *Malus *ortholog. All RT-qPCR primers for studied genes were designed with Primer Express (Applera Italia, Monza, Italy) software (Additional File 6).

### Analysis of VOCs

Frozen fruit tissue (15 g) was ground in liquid N_2 _into a fine powder. The volatile components were extracted with 100 ml methyl *tert*-butyl ether (MTBE) by vigorous shaking on a shaker apparatus overnight. *Iso*-butyl benzene (5 μg) was added as internal standard. The upper MTBE layer was separated, dried with sodium sulfate, and concentrated to a volume of 0.5 ml under a N_2 _stream. Samples were kept at 4°C until analysis. A 1-μl aliquot of the concentrated MTBE extract was injected into a GC-MSD (splitless mode). Results were an average of 6-8 replicate measurements.

GC-MS analysis was carried out using an Agilent GC-MSD system (CA, USA) EI Scan, equipped with a RTx-5sil MS column (injector temperature: 250°C; detector temperature: 280°C, 70 eV). Oven temperature profile was: 50 °C (1 min), 5° C/min to 280° C, 280° (5 min). Gas flow: 0.8 ml/min. Mass range: 41 to 500 *m*/*z*. Compound identification was performed by comparing their relative retention indices and mass spectra with those of authentic standards or with those found in the literature and supplemented with NIST 98 and QuadLib 1607 GC-MS libraries. A mixture of straight-chain alkanes (C7-C23) was injected into the column under the above-mentioned conditions for retention index calculation [53].

### Isolation and HPLC analysis of carotenoids

Plant material was homogenized in the presence of methanol and extracted three-times with MeOH. The methanolic extracts were combined and the carotenoid content of this solutions was transferred in a separatory funnel into toluene:hexane (1:1) mixture; evaporated and dissolved in diethyl-ether. After methanolic extractions, plant materials were then extracted once with diethyl-ether. The resulting extracts (ethereal solution of methanolic extracts + ethereal extract) were combined and saponified in a heterogeneous phase with 30% KOH/MeOH overnight (the 30% KOH/MeOH solution was layered on the lower part of the ethereal total extracts). After this process the reaction mixture was washed to alkali-free in a separatory funnel, dried over anhydrous Na_2_SO_4_, evaporated and the residues were dissolved in EtOH preparing the corresponding stock solutions for quantitative analysis. All of these procedures were carried out in semi-darkness and in N_2_-atmosphere [54-56].

The quantitative UV/VIS spectra of the corresponding stock solutions were recorded by a Jasco V-530 spectrophotometer. The total carotenoid content of the samples was calculated according to the Lambert-Beer's law (average molar extinction coefficient: 100,000; average molar mass of carotenoids: 600). The HPLC separation was performed with a Dionex Softron instrument (Germering, Germany) equipped with a Dionex P680 gradient pump, a Dionex PDA-100 detector and an end-capped C18 column (250 × 4.6 mm internal diameter; Merck LiChrospher 100 RP-18; 5 μm) thermostated at 22 ˚C. Elution was performed using 12% H_2_O/MeOH (A), MeOH (B), 50% Acetone/MeOH (C) at a flow rate of 1.25 ml min^-1^. The gradient program was: 100% A (0-2 min); 80% A and 20% B (2-10 min); 50% A and 50% B (10-18 min); 100% B (18-27 min); 100% C (27-34 min); 100% B (34-43 min); 100% A (43-56 min). Data acquisition was performed at 450 nm detection wavelength by Chromeleon 6.70 software.

### Statistical analyses

GC-MS data were submitted to principal component analysis (PCA) using Systat 11 software (http://www.systat.com). The data set was made up of data from eight repetitions of each ripening stage of RH and RHB. The variable set was made of the major 41 volatile aroma compounds. PCA involves a mathematical procedure that transforms a number of possibly correlated variables into a smaller number of uncorrelated variables called principal components. The first principal component accounts for as much of the variability in the data as possible, and each succeeding component accounts for as much of the remaining variability as possible. Gene expression data were submitted to hierarchical clustering analysis (HCA) using Systat 11. The HCA data set was made up from the means from independent experiments, for every stage of fruit development, of both genotypes. Means from independent RT-qPCR experiments were subjected to one-way ANOVA and Tukey's pairwise comparisons, and fruit quality data to *t *test, carried out using PAST (http://folk.uio.no/ohammer/past/).

## Authors' contributions

FB did the samplings, organized the work, carried out molecular experiments and statistical analyses, and contributed to manuscript writing; EB carried out VOC extractions and GC-MS analyses; FM designed and participated to molecular experiments and data analysis, and contributed to manuscript writing; GH and ET performed carotenoid extractions, HPLC and data analyses; GG contributed early research design and molecular data, and to manuscript writing; AL and ST tutored FB, helped in field experiments and data analysis, and contributed to manuscript writing; EL designed GC-MS analyses and interpreted their data, and contributed to manuscript writing; CR designed and coordinated the research, contributed to statistical analyses and wrote the manuscript. All authors read and approved the final manuscript.

## Supplementary Material

Additional File 1 **Structures of the main carotenoids identified in RHB and RH fruits during ripening**. Carotenoid composition is reported in Table 1. Click here for file

Additional File 2 **Major quality traits of RHB and RH fruits measured at S4 stage**. SSC: soluble solids content (expressed in °Brix). TA: titratable acidity (expressed in meq NaOH/l). Firmness was measured with an 8-mm diameter tip (expressed in kg/cm^2^). Skin color parameters a* (red chromatic coordinate), b* (yellow chromatic coordinate) and L* (brightness) were recorded at two fruit cheeks (opposite equatorial points). Values are average measurements of ten representative fruits. Different letters indicate significant differences among mean values (*t *test; *p *≤ 0.05). Click here for file

Additional File 3 **Hierarchical clustering analysis of carotenoid gene expression in RH and RHB genotypes**. A: joint analysis of RHB and RH data. B: RHB data only. C: RH data only. Each cell corresponds to the relative expression value (Log-transformed) according to the color scale on the right. For enzyme abbreviations and fruit development stages, see text and Methods, respectively. Click here for file

Additional File 4 **Total VOC content in RHB and RH mesocarp during fruit ripening**. RH: solid black squares. RHB: open squares. Values ± SD are in ng/g fresh weight. Click here for file

Additional File 5 **Accumulation patterns of identified norisoprenoids in RHB and RH mesocarp during fruit ripening**. RH: solid black symbols. RHB: open symbols. Values are in ng/g fresh weight. Click here for file

Additional File 6 **Sequences of RT-qPCR primers used in this work**. for experimental conditions, see Methods. Click here for file
