# Supplementary figures and images for: Study of 'Redhaven' peach and its white-fleshed mutant suggests a key role of CCD4 carotenoid dioxygenase in carotenoid and norisoprenoid volatile metabolism
Source: BMC Plant Biol. 2011 Jan 26;11:24. doi: 10.1186/1471-2229-11-24 (PMC3045293; doi:10.1186/1471-2229-11-24)

## Slide 1
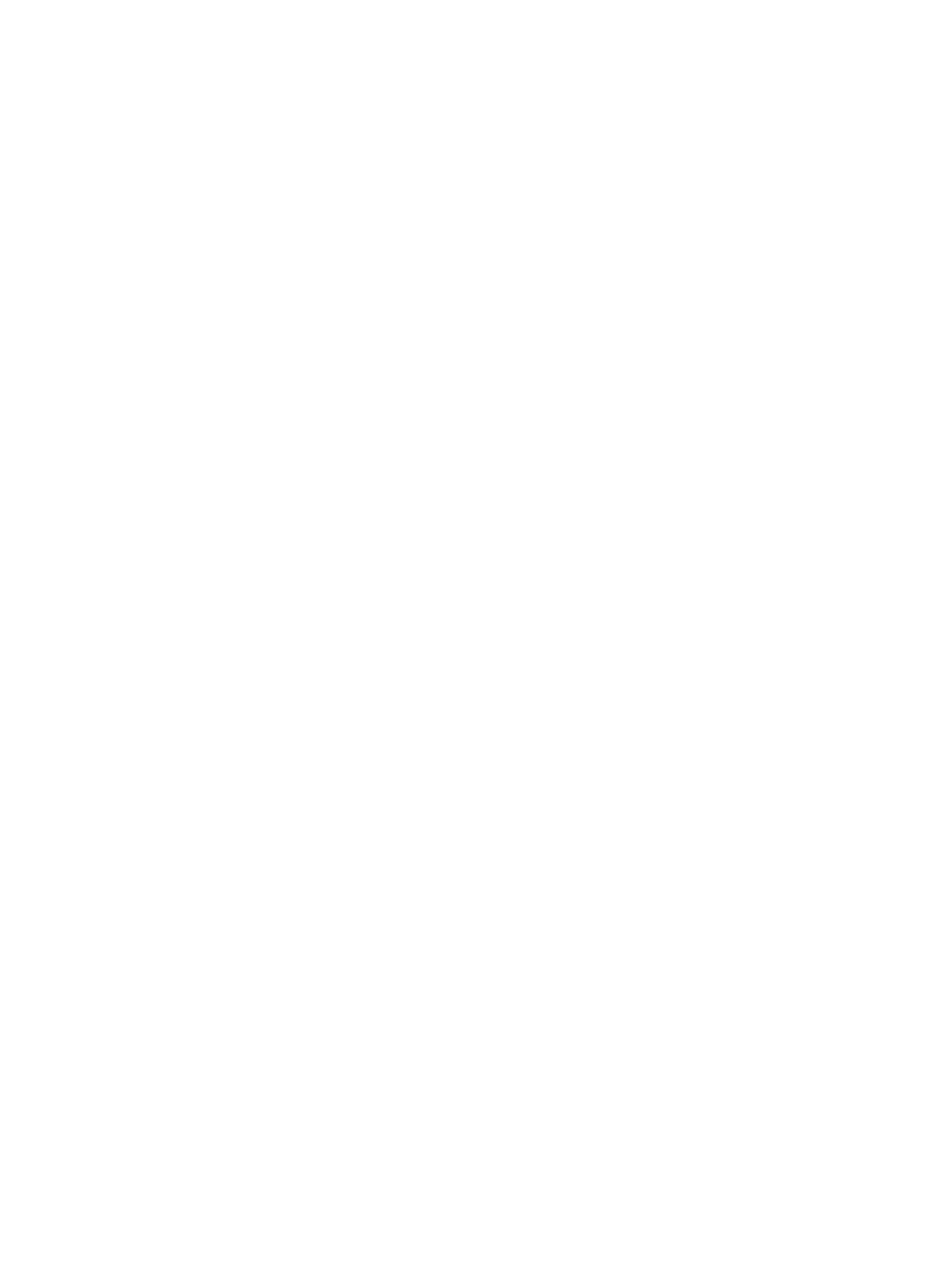

Supplement: Additional File 1 — Structures of the main carotenoids identified in RHB and RH fruits during ripening. Carotenoid composition is reported in Table 1. [file 1471-2229-11-24-S1.PPT]

## Slide 1
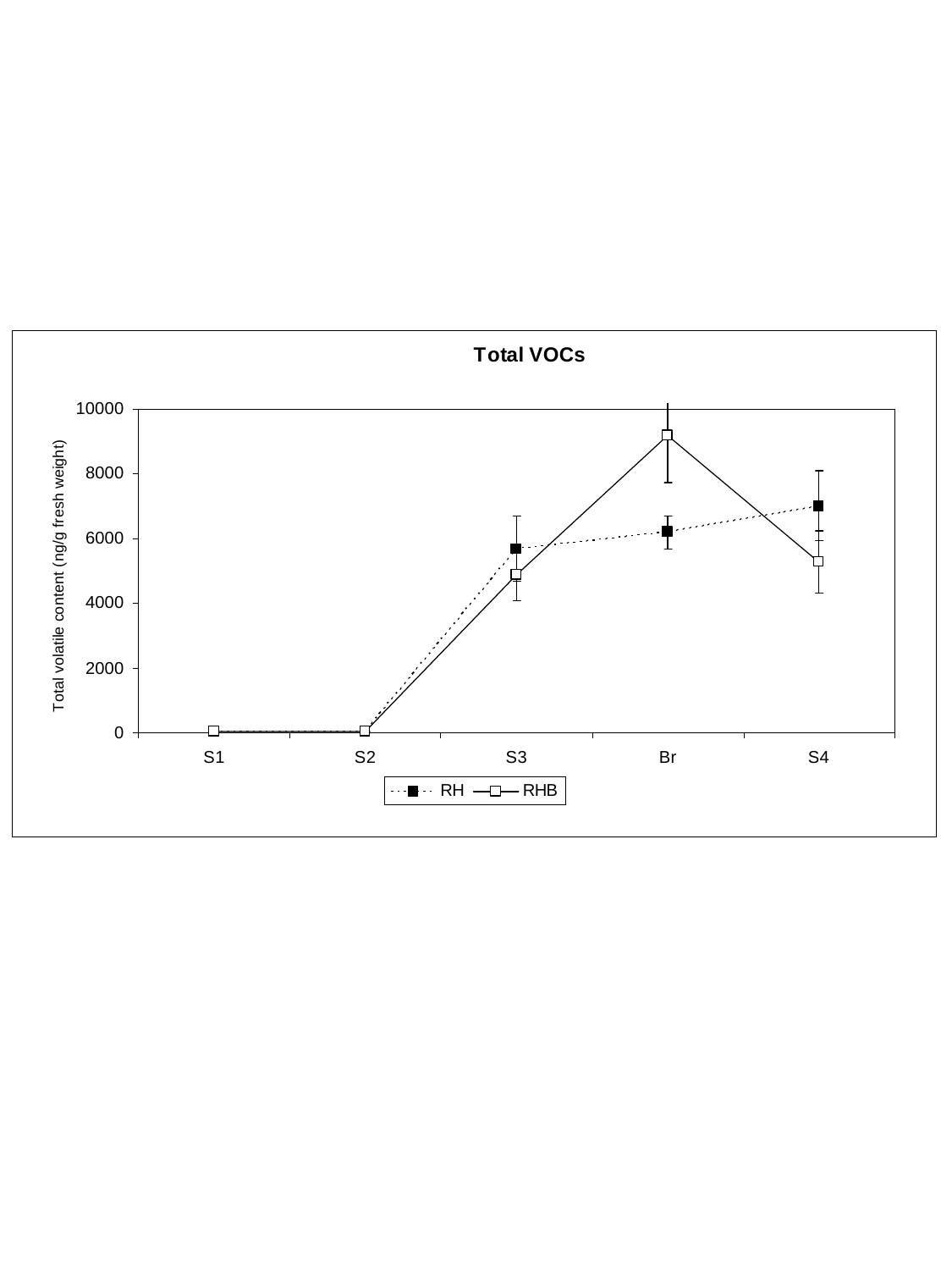

Supplement: Additional File 4 — Total VOC content in RHB and RH mesocarp during fruit ripening. RH: solid black squares. RHB: open squares. Values ± SD are in ng/g fresh weight. [file 1471-2229-11-24-S4.PPT]

## Slide 1
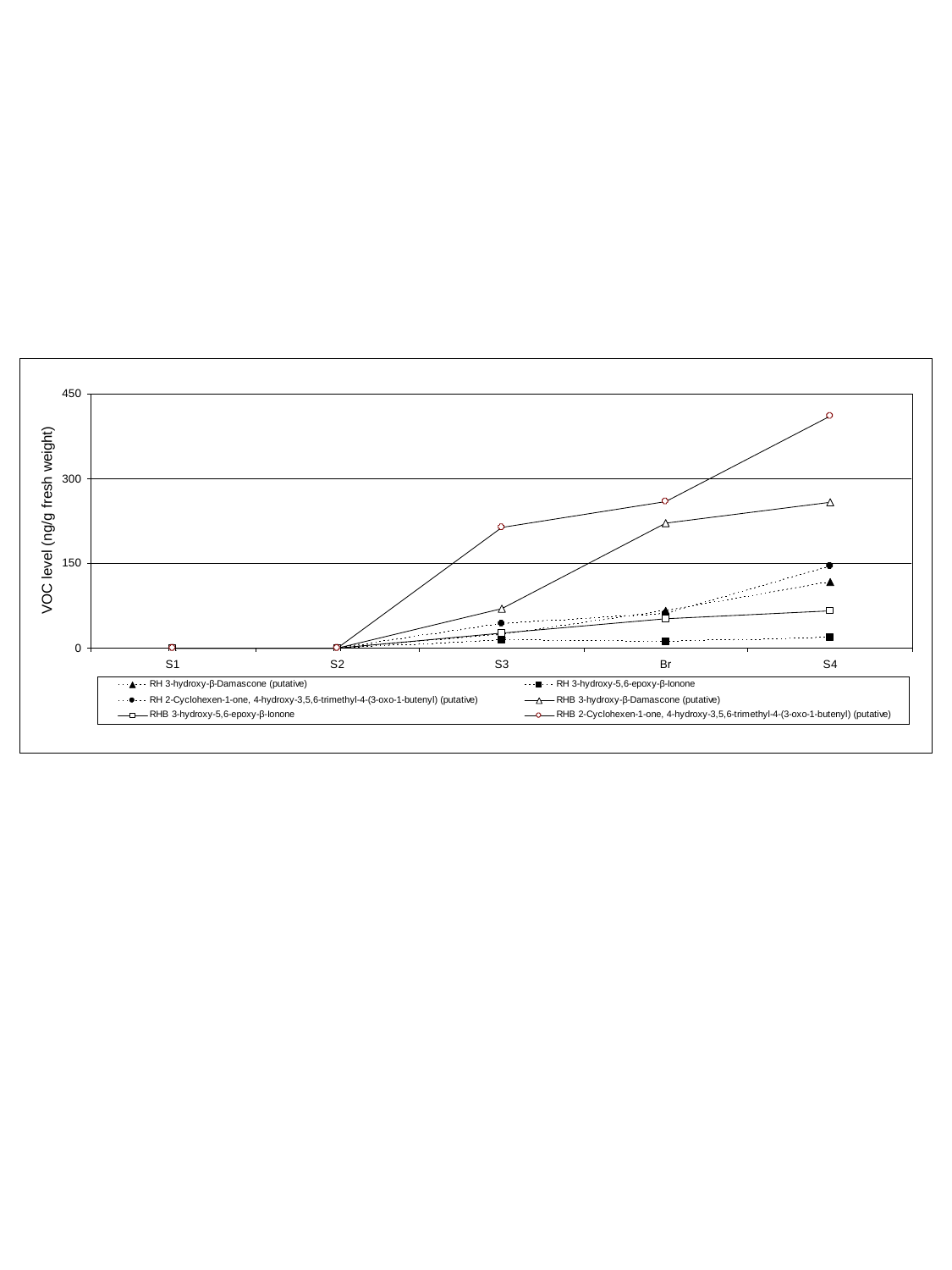

Supplement: Additional File 5 — Accumulation patterns of identified norisoprenoids in RHB and RH mesocarp during fruit ripening. RH: solid black symbols. RHB: open symbols. Values are in ng/g fresh weight. [file 1471-2229-11-24-S5.PPT]
