# Supplementary material for: Study of 'Redhaven' peach and its white-fleshed mutant suggests a key role of CCD4 carotenoid dioxygenase in carotenoid and norisoprenoid volatile metabolism
Source: BMC Plant Biol. 2011 Jan 26;11:24. doi: 10.1186/1471-2229-11-24 (PMC3045293; doi:10.1186/1471-2229-11-24)
Supplement: Additional File 2 — Major quality traits of RHB and RH fruits measured at S4 stage. SSC: soluble solids content (expressed in °Brix). TA: titratable acidity (expressed in meq NaOH/l). Firmness was measured with an 8-mm diameter tip (expressed in kg/cm2). Skin color parameters a* (red chromatic coordinate), b* (yellow chromatic coordinate) and L* (brightness) were recorded at two fruit cheeks (opposite equatorial points). Values are average measurements of ten representative fruits. Different letters indicate significant differences among mean values (t test; p ≤ 0.05). [file 1471-2229-11-24-S2.DOC]

**Additional File 2.** **Major quality traits of RHB and RH fruits measured at S4 stage.**

|  | | | | Skin color | | |
| --- | --- | --- | --- | --- | --- | --- |
| Genotype | SSC | TA | Firmness | a* | b* | L* |
| RH | 10.9^a^±1.5 | 77.9^a^ ±13.5 | 3.2^a^±1.2 | 29.2^a^±3.7 | 15.5^a^±2.8 | 38.3^a^±3.8 |
| RHB | 9.2^b^±1.5 | 83.8^a^±19.4 | 3.8^a^±0.5 | 25.0^a^±3.2 | 13.5^a^±1.7 | 39.5^a^±2.3 |

SSC: soluble solids content (expressed in °Brix). TA: titratable acidity (expressed in meq NaOH/l). Firmness was measured with an 8-mm diameter tip (expressed in kg/cm^2^). Skin color parameters a* (red chromatic coordinate), b* (yellow chromatic coordinate) and L* (brightness) were recorded at two fruit cheeks (opposite equatorial points). Values ± SD are average measurements of ten representative fruits. Different letters indicate significant differences between mean values (*t* test; *p* ≤ 0.05).
