# Supplementary material for: Study of 'Redhaven' peach and its white-fleshed mutant suggests a key role of CCD4 carotenoid dioxygenase in carotenoid and norisoprenoid volatile metabolism
Source: BMC Plant Biol. 2011 Jan 26;11:24. doi: 10.1186/1471-2229-11-24 (PMC3045293; doi:10.1186/1471-2229-11-24)
Supplement: Additional File 3 — Hierarchical clustering analysis of carotenoid gene expression in RH and RHB genotypes. A: joint analysis of RHB and RH data. B: RHB data only. C: RH data only. Each cell corresponds to the relative expression value (Log-transformed) according to the color scale on the right. For enzyme abbreviations and fruit development stages, see text and Methods, respectively. [file 1471-2229-11-24-S3.PPT]

## Slide 1
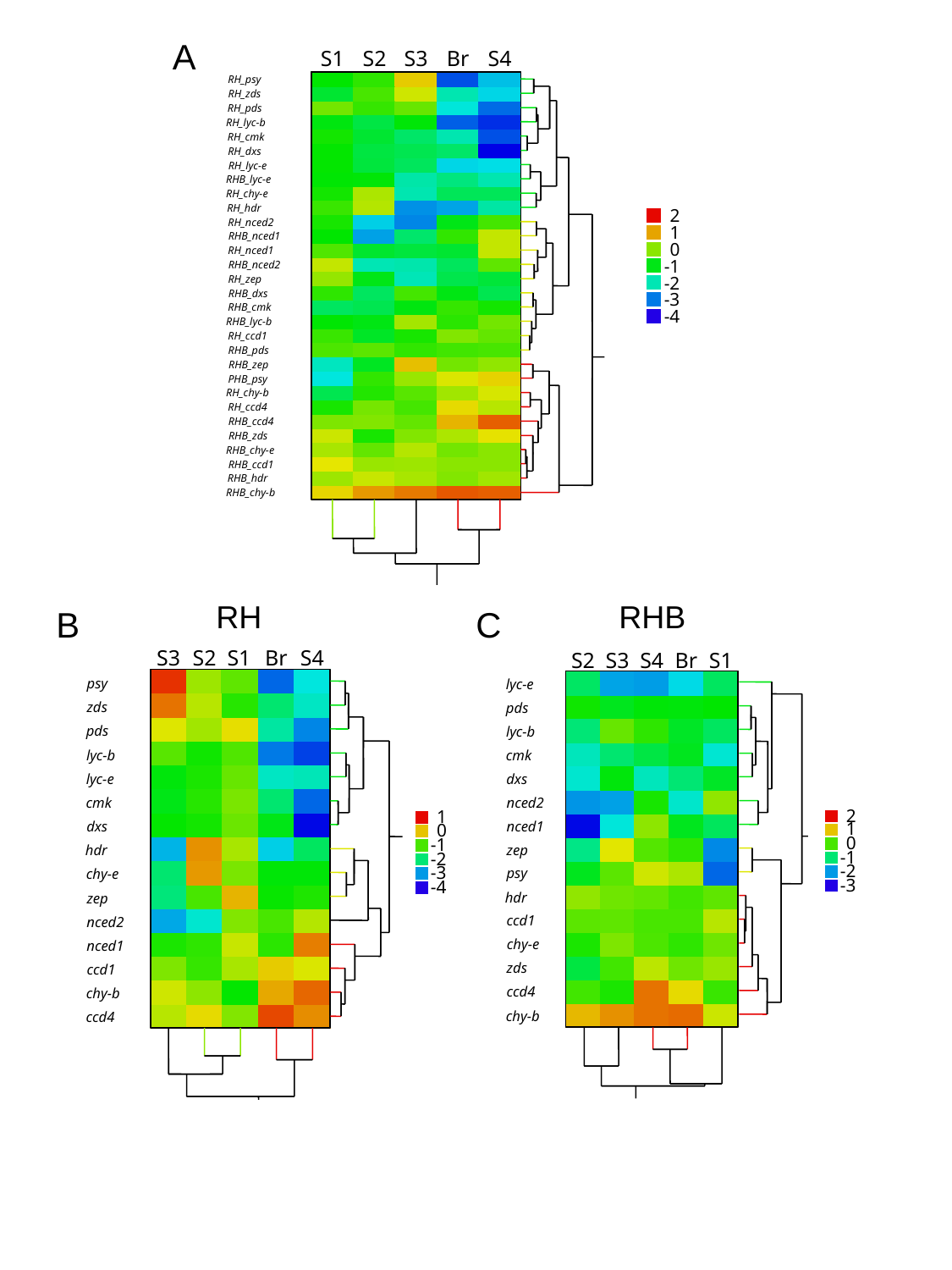

A
S1
S2
S3
Br
S4
RH_psy
RH_zds
RH_pds
RH_lyc-b
RH_cmk
RH_dxs
RH_lyc-e
RHB_lyc-e
RH_chy-e
RH_hdr
2
RH_nced2
1
RHB_nced1
0
RH_nced1
-1
RHB_nced2
-2
RH_zep
RHB_dxs
-3
RHB_cmk
-4
RHB_lyc-b
RH_ccd1
RHB_pds
RHB_zep
PHB_psy
RH_chy-b
RH_ccd4
RHB_ccd4
RHB_zds
RHB_chy-e
RHB_ccd1
RHB_hdr
RHB_chy-b
RH
RHB
B
C
S3
S2
S1
Br
S4
S2
S3
S4
Br
S1
psy
lyc-e
zds
pds
pds
lyc-b
lyc-b
cmk
lyc-e
dxs
nced2
cmk
2
1
nced1
dxs
1
0
0
-1
zep
hdr
-1
-2
-2
-3
psy
chy-e
-3
-4
hdr
zep
ccd1
nced2
chy-e
nced1
zds
ccd1
ccd4
chy-b
chy-b
ccd4
