# Supplementary material for: Study of 'Redhaven' peach and its white-fleshed mutant suggests a key role of CCD4 carotenoid dioxygenase in carotenoid and norisoprenoid volatile metabolism
Source: BMC Plant Biol. 2011 Jan 26;11:24. doi: 10.1186/1471-2229-11-24 (PMC3045293; doi:10.1186/1471-2229-11-24)
Supplement: Additional File 6 — Sequences of RT-qPCR primers used in this work. for experimental conditions, see Methods. [file 1471-2229-11-24-S6.DOC]

**Additional File 6.** **Sequences of RT-qPCR primers used in this work.**

| Gene | Sequence | Forward primer | Reverse primer |
| --- | --- | --- | --- |
| *dxs* | Ref. [51] | GATTGCTTTGGCACTGGTCA | CCCCAAGCCTGCTGAGATAGT |
| *cmk* | Ref. [51] | ATAAAGCCCCAACAGGCATG | TTGAAGGCGCTTGTAAACTTCA |
| *hdr* | BU042033 | ACCACTTTGTCAGCTTCAACACA | CTTGTCGCTCTTGAGTCGCA |
| *psy* | DT454972 | GATAGGTGGGCAGTCGAGGTT | TCAAATGGACGACCTTGGAAA |
| *pds* | AJ825914 | GGACAAACAAGGGTGGCTGA | CTTCAAGGTTTTTCGTCACCG |
| *zds* | DY650686 | GTCCAAAGGTGGCACACGA | GCAACAGGATCCCACATCCT |
| *lyc-b* | BU046272 | CCTGGTCTGGTGCTGTTGTG | AGATCCTTTTTCGTCCTGCTCA |
| *lyc-e* | Ref. [51] | AAGCATGGAACACCCTCTGG | AGAATGCTCTTTGGCGCTTC |
| *zep* | DY644253 | CCAAATATGGGTCAAGGTGGAT | TTTCGCTACTTTTCTTCCATGCT |
| *chy-b* | DW346978 | GCTCGAGGAAGCTCTGTTTCA | CTTGGGCTTTTTCTGTGCAATT |
| *chy-e* | DY640014 | TCCAAGCATCCTTCGCTTTTT | TCCAGCAACCAACATAGACAGAA |
| *ccd1* | DW350114 | ATCAGATGGTTCGACCTTCCAA | GGCGGCATGTGATTAAAACAAT |
| *ccd4* | DT455068 | GGCTAGAGAGCCCGAGAATC | GAGGAGACTTGGCATCCATC |
| *nced1* | DY638454 | ATGGCTGCTCTTGGAAAAGC | AGCCCATAGGATCCACTAGA |
| *nced2* | DY651361 | CTGGAAGCGACGGTGAAATT | GCTATGCCTGATTTGCCAAGTC |
| *rps28* | AJ012655 | GCATGCTGTGGTTGTGAAAGTT | CCGATTCTGGTCATCCAAAAAC |

Sequence references and NCBI accessions used for primer design are listed. For RT-qPCR experimental conditions, see Methods.
